# Environmental and biological monitoring in the workplace: A 10-year South African retrospective analysis

**DOI:** 10.12688/aasopenres.12882.2

**Published:** 2019-07-23

**Authors:** Puleng Matatiele, Lerato Mochaki, Bianca Southon, Boitumelo Dabula, Poobalan Poongavanum, Boitumelo Kgarebe

**Affiliations:** 1 Analytical Services, National Institute for Occupational Health, Johannesburg, Gauteng, 2000, South Africa; 2 African Academy of Sciences, Nairobi, Kenya

**Keywords:** Analytical Services, chemical exposure, environment, monitoring, heavy metals

## Abstract

This report is an overview of requests for biological and environmental monitoring of hazardous chemicals, submitted to the National Institute for Occupational Health, Analytical Services Laboratory for testing from the years 2005 to 2015. The report discusses the nature of tests requested and implications for workers’ health and environment, as well as potential impact of the uncertainties associated with monitoring of hazardous chemicals. This is a retrospective, descriptive, qualitative and quantitative audit of all samples received and tests performed retrieved from records of analysis by the laboratory. The study sample consisted of 44,221 samples. The report indicates that throughout the interrogation period the demand for biological monitoring was higher than that for environmental monitoring, with more requests for toxic metals than organic pollutants. Toxic metal testing was highest for mercury, followed by manganese, lead, aluminium and arsenic. The highest number of tests for organic pollutants was conducted for pesticides followed by toluene and xylene. The study has also revealed that the scope of tests requested is rather narrow and does not reflect the broad spectrum of **South Africa’s industrial diversity. **Having identified possible reasons for underutilization, a number of reforms that could enhance the laboratory’s performance have been addressed.

## Introduction

Humans are exposed to hazardous chemicals in a variety of ways; mainly through diet and through the air that we breathe (indoor, outdoor and occupational). Occupational exposure can occur through inhalation, absorption through the skin or ingestion, with the inhalation of vapours, dusts, fumes or gases being the route of highest exposure ^1^. Both biological and environmental monitoring can help in assessment of exposure to specific chemicals, characterization of exposure pathways and potential risks and their mitigation, and thus serve as elements of health surveillance that can be used in the assessment of the risks to health as an integral part of occupational and environmental health and safety programmes. Thus, the three-pronged prevention of diseases due to toxic agents in the general or occupational environment involves both environmental and biological monitoring, as well as health surveillance ^2^. In the occupational context, environmental monitoring entails characterization and monitoring of the quality of the environment in preparation for environmental impact assessment ^3, 4^. As a result, environmental monitoring is critical to understanding whether the quality of the environment is getting better or worse, and allows for the removal of a worker from a contaminated environment before adverse health effects are experienced. Biological monitoring in the workplace involves the detection of biomarkers in biological samples (e.g., breath, urine, blood, hair, etc.) from workers, and the comparison to reference values ^5, 6^. Guidelines for chemical monitoring strategies have established that monitoring is necessary if there is reason to believe that a hazard exists or may develop in the workplace ^7^. Thus, monitoring and surveillance are valuable tools enabling identification and tracking of exposures to hazards in the environment and their related health implications. It is through the results of monitoring and surveillance programs that it becomes possible for authorities to make sound and effective public and environmental health policies and interventions, as well as enabling employers to measure the efficacy of control measures.

The National Institute for Occupational Health (NIOH) in South Africa (SA) has a well-established analytical chemistry laboratory (commonly referred to as Analytical Services) that specialises in hazardous chemical exposure analyses. The overall goal of the laboratory is to promote effective environmental and biological monitoring and surveillance of existing and emergent chemical hazards related to workplace chemicals and to environmental quality. As already indicated, quantifying exposure levels and generating science-based information is necessary to identify risks and inform risk management. The laboratory offering consists of different techniques of both well-established (accredited) and other novel technologies.

The current review focuses on the request for hazardous (organic and inorganic) chemicals analysis in Analytical Services of the NIOH as markers of biological and environmental exposure in the workplace. The number and type of tests requested from this laboratory shed light on the demand for biological and environmental monitoring of these chemicals of concern in workplaces. Hence the results of this study will bring to light whether the number and type of tests requested reflect SA’s mineral riches and industrial diversity where human exposure to these chemicals is highly possible.

## Methods

### Data source

This was a retrospective and descriptive study of the number of samples received and tests performed retrieved from records of analysis by the laboratory for the years 2005 to 2015. The study sample consisted of 30,399 samples analysed for toxic metals and 13822 samples for organic pollutant exposure. All samples for organic pollutants analysis were biological matrices (urine, blood and plasma), whereas samples for analysis of toxic metals comprised of both human (urine, blood, serum, plasma and tissue), and a variety of environmental matrices (water, dust, filters, paint, ink, traditional medicine concoctions, etc.). In total there were 40,931 and 3,290 human and environmental samples, respectively. These samples were from a variety of industries ranging from mining, petrochemicals, motor industry, agriculture, waste processing and the army. The data was entered onto an Excel spreadsheet. Tables and graphs were generated by number of tests per toxic metal, matrix type and organic pollutant.

### Ethics

As a clinical chemistry laboratory, Analytical Services is accredited to both ISO 15189 and ISO/IEC 17025 by the South African National Accreditation System, (registration number M0276). The laboratory follows principles of Good Clinical Practice, which is an international ethical and scientific quality standard for designing, conducting, recording and reporting studies that involve the participation of human subjects. Compliance with the standard provides public assurance that the rights, safety and wellbeing of study subjects are protected, consistent with the principles of the Declaration of Helsinki ^8, 9^.

## Results

**Figure 1.**
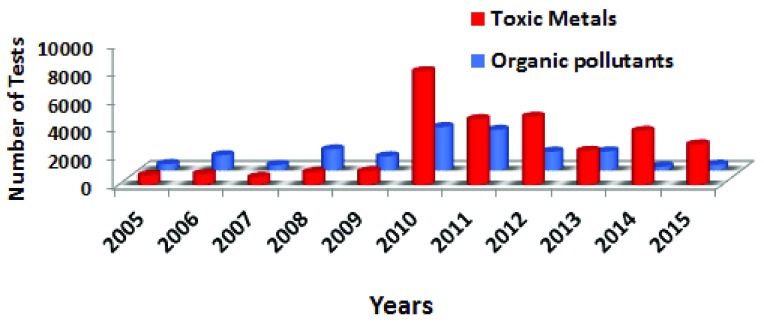
The total number of tests for both toxic metals and organic pollutants done per year throughout the years 2005 to 2015 at NIOH.

**Figure 2.**
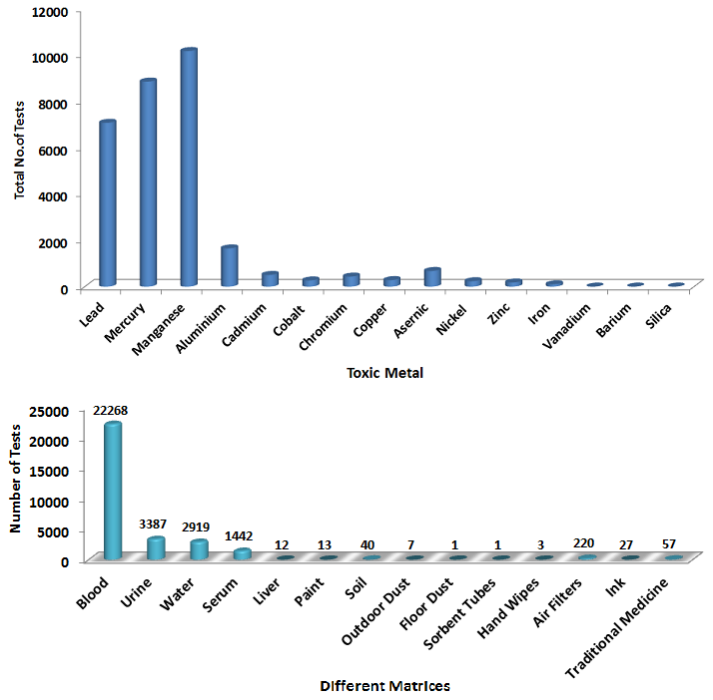
The total number of tests performed for environmental and biological monitoring of ( **a**) toxic metals in ( **b**) various matrices at NIOH from years 2005 to 2015.

**Figure 3.**
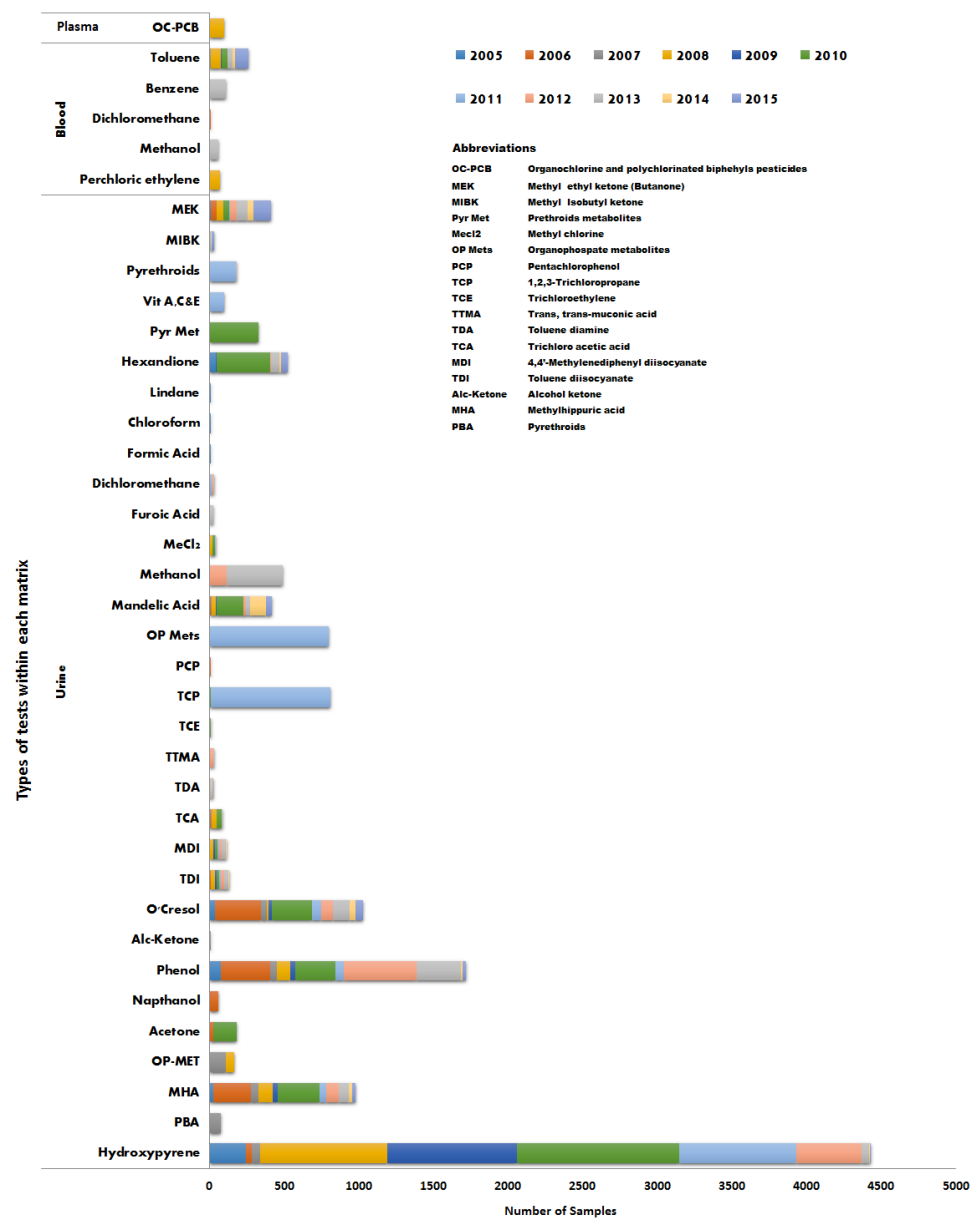
The total number of tests performed for environmental and biological monitoring of organic pollutants at NIOH from years 2005 to 2015.

Table 1 shows the total number of tests performed for the analysis of both toxic metals and organic pollutants per year for the period 2005 to 2015 at NIOH, the results of which are available on OSF ^10^. Generally, there were more requests for analysis of toxic metals than for that of organic pollutants ( Figure 1). In addition, analysis of both toxic metals and organic pollutants exposure grew steadily from 2005 to 2010, after which the laboratory saw a decline in the number of test requests. With regard to individual toxic metals tests, Figure 2a shows that the demand for inorganic mercury testing had the highest frequency, followed by manganese and lead. The most common matrix tested was blood followed by urine, water, and serum ( Figure 2b). Organic pollutant monitoring results ( Figure 3) show that the highest number of tests was conducted for pesticides, followed by phenol, toluene and xylene. These pollutants are as a result of activities mainly from the agriculture (pesticides) and petrochemical/motor and mining (phenol, toluene and xylene) industries. Even though no data has been shown in this study, NIOH has also received requests for toxic chemical testing in a wide variety of samples, including nails, hair and unidentified powders and liquids. Also not shown are requests from clinicians for therapeutic drug monitoring and forensic toxicology assessment.

**Table 1.** The total number of toxic metals and organic pollutants tests done per year throughout the years 2005 to 2015 at NIOH.

Year	2005	2006	2007	2008	2009	2010	2011	2012	2013	2014	2015	Total
Toxic Metals	659	743	543	874	941	8066	4658	4825	2403	3824	2863	30399
Organic pollutants	457	1084	393	1503	1014	3093	2889	1340	1362	281	406	13822
Total	1116	1827	936	2377	1955	11159	7547	6165	3765	4105	3269	44221

## Discussion and conclusions

Assessment and characterization of the body-burden of hazardous chemicals and the potential health risks thereof is a key strategy for providing a scientific basis for prevention via exposure reduction and motivating action especially in occupational settings. The increase in bio-monitoring versus environmental monitoring, as seen in our results, confirms some literature findings, which report that exposure assessment has shifted from pollutant monitoring in air, soil, and water towards personal exposure measurements and bio-monitoring ^11^. The decline in the number of tests requested from NIOH Analytical Services laboratory in latter years is noted. The decline is suggestive of an underutilized resource, which could have been as a result of various reasons, including:

- Samples received being unlabelled, wrongly labelled or not collected properly thereby resulting in rejection by the laboratory ^12^, hence why as one of its key performance areas the Analytical Services laboratory conducts upon request free training on sample collection, handling and transportation of samples in the field of occupational and environmental health.
- Previous regular users could have been unhappy and therefore left due to reasons such as long turn-around times, market-related pricing of tests, etc. Also, the unavailability of some test methods, as a result of the lack of suitable equipment and/or the relevant expertise could drive away clients. Consequently, NIOH has prioritized the purchase of state-of-the-art equipment, which is the gold standard equipment used for the services required. The use of these technologies and techniques, either individually, or in combination, has become essential in modern laboratory and environmental medicine.
- Lack of marketing; potential users could have been unaware of NIOH as a service provider for laboratory testing for the purposes of environmental and biological monitoring and surveillance so that there was no growth in numbers of new requisitions. However, the growing number of current requests for testing (though not shown) indicates growing awareness for this specialized service which is the only one in the country for public service.

**Figure 4.**
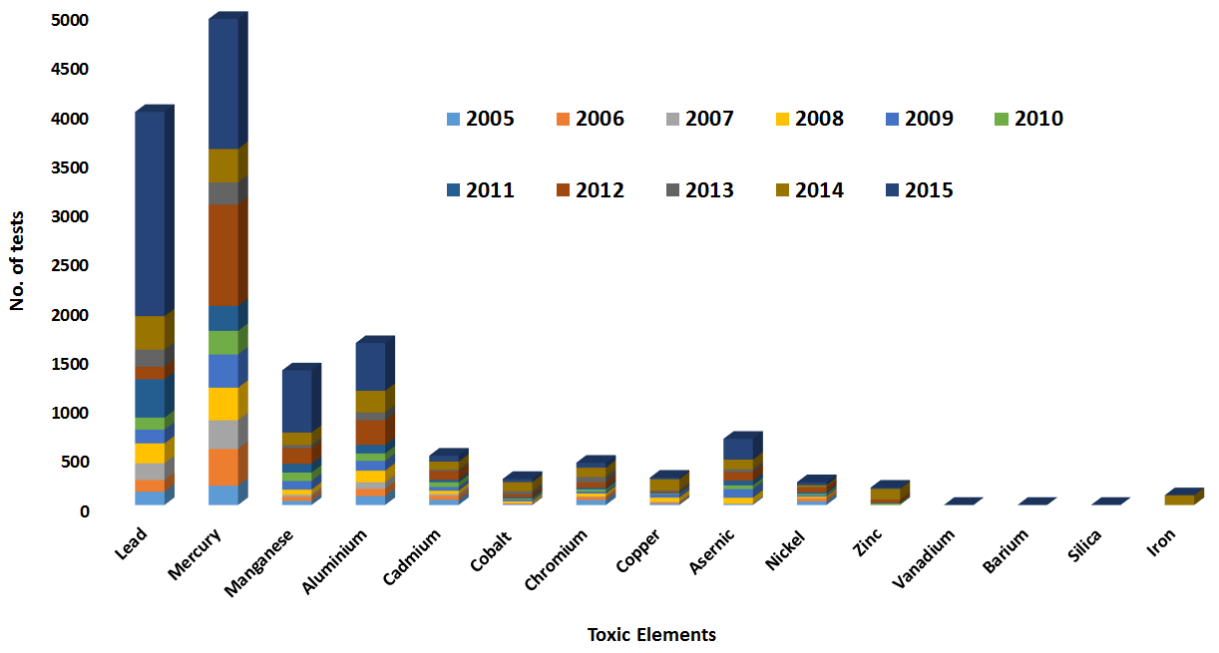
The total number of tests performed per year for toxic metals at NIOH from years 2005 to 2015.

Notably, the rises and dips in the frequency of requests correlate strongly with environmental disaster occurrences and awareness campaigns, and their decline, in the country. Naturally, environmental disasters and work incidents pique the interest of various groups, namely regulators, environmentalists and workers’ rights groups, thereby putting pressure on the government and companies associated with these incidents to take action to monitor both the affected workers and the environment. For example, one international chemicals company operating in SA made headlines in the late 1990s when it was found guilty of having exposed some of its workers and nearby communities to massive quantities of mercury as well as keeping stock-piled mercury waste that had started to leach into soil and water bodies ^13^. As Figure 4 also shows, subsequent environmental monitoring requirements saw a peak in requests for mercury analysis ^14– 19^. Similarly, several studies conducted in SA, probably in the wake of global lead poisoning prevention campaigns, have revealed the sources and potential risk factors associated with human lead poisoning, thereby stimulating awareness leading to monitoring in highly susceptible groups, including children or workers in high risk work areas such as lead mines, shooting ranges, battery manufacturing, painting and many others ^20– 25^. In a similar manner, manganism came to the fore in 2007 when several cases of suspected chronic exposure to manganese had been detected at a ferromanganese smelter ^26^. A similar pattern was observed for exposure to arsenic and several other heavy metals ^27– 29^.

This study has also revealed that the scope of tests requested is quite limited and does not reflect the broad spectrum of SA’s mineral riches. SA’s main raw materials mined are gold, diamonds, platinum, chromium, vanadium, manganese, uranium, iron, coal and copper. Scientific studies have confirmed that mining of any of these minerals presents health hazards; hence human exposure to them should be monitored as well as the environment in which their use occurs ^30^. However, the spectrum of tests requested for biological and environmental monitoring at the Analytical Services is not representative of this variety of raw materials. Perhaps other workplaces make use of alternative private laboratories for their monitoring, which if not, could be indicative of lax monitoring (or no monitoring at all) with regard to exposures associated with the mining and use of these raw materials. For example, in SA it is common knowledge that artisanal mining, and small scale and illegal mining are thriving, especially at abandoned/disused underground mines ^31^. Workers in these environments are exposed to a wide variety of minerals, including lead, silica, gold, manganese, and platinum. Mining regulatory authorities do not monitor activities in these types of mining environments; and therefore workers and their associated health risks cannot be monitored and measured ^32, 33^.

Thus, having identified the reasons for underutilization of such a valuable resource, the NIOH started working on action-oriented steps to address some of these problems; hence, the above outline of a number of reforms that have been implemented and have enhanced the laboratory’s performance. In a similar manner, having analysed the trends above, the NIOH continues to work on action-oriented steps to address some of the identified gaps. For example, as the awareness of exposures and the science of measurement and associated diseases grow, stronger engagement with stakeholders (regulators, labour and business) and provision of resources to upgrade the existing laboratories and engage in contract research focusing on biological and environmental monitoring of the impact of technology has resulted in the upcoming acquisition of state-of-the-art equipment.

This study is, however, not without its own limitations. Lack of extensive interrogation of data in the study leaves many questions unanswered. For example, the number of samples submitted for testing does not necessarily imply unacceptable exposure levels to the particular hazardous chemical under scrutiny. While this could be one reason an employer would deem it unnecessary and uneconomical to carry out continuous monitoring, neither is testing many individual workers an indication of a safe working environment. The uncertainty regarding reasons why monitoring was initiated but later abruptly terminated could have been dealt with better by sending out questionnaires to the tests requesters. It is thus difficult with the current information to determine if there was compliance with Occupational Exposure Limit (OEL) values or not. Hence, this work only forms the basis upon which an extensive future study will be conducted which will consider the relevant reference values to determine safety conditions of the various work environments. Nevertheless, Analytical Services provides an invaluable service that should be utilized to its full capacity if workers’ health in the region is to improve. In fact, thousands of workers still are becoming ill and dying as a result of exposure to hazardous chemicals, because they are being exposed to levels of chemicals that are not necessarily illegal, but are not safe ^34, 35^, hence why consistent monitoring should be encouraged.

## Data availability

**Complete data regarding the number of tests for each metal and organic compound** are available on OSF: http://doi.org/10.17605/osf.io/fh8z7 ^10^. Data are displayed per year and consolidated.
