# Supplementary material for: Environmental and biological monitoring in the workplace: A 10-year South African retrospective analysis
Source: AAS Open Res. 2019 Jul 23;1:20. Originally published 2018 Jul 16. [Version 2] doi: 10.12688/aasopenres.12882.2 (PMC7194147; doi:10.12688/aasopenres.12882.2)
Supplement: Peer review history

# Peer review: Reviewer response for version 2

**DOI:** 10.21956/aasopenres.14065.r27087

**Published:** 2019-08-12

**Authors:** Jolanta Skowroń

**Affiliations:** 1 Central Institute for Labour Protection, National Research Institute, Warsaw, Poland

I agree with the authors' explanations to my comments. I suggest to publish the article in Version 2.

I confirm that I have read this submission and believe that I have an appropriate level of expertise to confirm that it is of an acceptable scientific standard.

# Peer review: Reviewer response for version 1

**DOI:** 10.21956/aasopenres.13949.r26936

**Published:** 2019-05-29

**Authors:** Jolanta Skowroń

**Affiliations:** 1 Central Institute for Labour Protection, National Research Institute, Warsaw, Poland

The title of the article by Matatiele *et al*. promises a lot, but what is involved is a pure analysis of the laboratory's activity that measures the concentrations of chemicals in the air of the work environment and in the biological material.

 More interesting for occupational health and safety would be information on the assessment of exposure to chemicals at workplaces with respect to applicable occupational exposure limits. The same problem applies to biological monitoring in the work environment, which in the EU now is becoming increasingly important in the assessment of exposure for example to carcinogens. The authors of the article have referred to reference values for both types of monitoring, but these are articles from 1995 and do not contain reference values.

 The number of tests and analysis carried out in the working environment does not prove safety conditions. Only information about compliance with OEL values or exceeding them is valuable for the OSH and employer services, because it requires the use of appropriate technical, technological or organizational measures to provide employees safety working conditions.

 Very interesting is the number of the analyses in biological material (blood, urine, breath, hair, etc.), but without the reference value it is difficult to interpret the results. Has biological monitoring been performed in employees for whom concentrations of e.g. metals in the work environment have been determined? If so, what should be done OSH service, if these values are exceeded and the concentrations in the working environment are below the limit values? Similarly, it was interesting to also consider the reverse situation.

 I hope that this article is just an introduction to undertake deeper analysis regarding working environment with particular emphasis on biological monitoring.

I confirm that I have read this submission and believe that I have an appropriate level of expertise to confirm that it is of an acceptable scientific standard, however I have significant reservations, as outlined above.

# Peer review: Reviewer response for version 1

**DOI:** 10.21956/aasopenres.13949.r26576

**Published:** 2018-10-01

**Authors:** Alastair W. M. Hay

**Affiliations:** 1 Department of Environmental Toxicology, School of Medicine, University of Leeds, Leeds, UK

This paper by  P Matatiele *et al* is suitable for indexing as it stands. It is well written and I could find no mistakes that require correction.

 The paper is an analysis of requests and tests carried out by the National Institute of Occupational Health’s Analytical Services laboratory over the decade 2005 to 2015. It is interesting to read about what was requested and when. The authors surmise on reasons for the decline in certain areas is also interesting.

 But for me this paper raises many other questions and it is something I would like both the authors and editors to consider.

 It is all very well to list requests, however, for practicing scientists the more interesting question is what did the tests show? It would help to know what the range of results were, what the mean was and how many exceeded the appropriate national limit? For those which exceeded limits were there any follow- up requests?

 What I am suggesting here is a much more extensive piece of work and perhaps material for a follow–on publication.

 Regarding the tests performed when results are expressed I would want to know the methods used, limits of detection and accuracy and reproducibility of methods.

 As for reasons for declining requests the authors cite various possibilities. This begs the question: Have the authors sent out any questionnaire to users to get feedback about the service and to ascertain where changes are needed? They refer to long periods to generate data. How long might these intervals be and what is being done to redress them? Is the new equipment they refer to any attempt to bridge some of these problems?

I confirm that I have read this submission and believe that I have an appropriate level of expertise to confirm that it is of an acceptable scientific standard.
